# Influence of Hand Tracking in Immersive Virtual Reality for Memory Assessment

**DOI:** 10.3390/ijerph20054609

**Published:** 2023-03-05

**Authors:** José Varela-Aldás, Jorge Buele, Irene López, Guillermo Palacios-Navarro

**Affiliations:** 1 Centro de Investigaciones de Ciencias Humanas y de la Educación—CICHE, Universidad Indoamérica, Ambato 180103, Ecuador; 2 SISAu Research Group, Facultad de Ingeniería, Industria y Producción FAINPRO, Universidad Indoamérica, Ambato 180103, Ecuador; 3 Department of Electronic Engineering and Communications, University of Zaragoza, 44003 Teruel, Spain

**Keywords:** immersive virtual reality, hand tracking, memory assessment, presence, usability, satisfaction

## Abstract

Few works analyze the parameters inherent to immersive virtual reality (IVR) in applications for memory evaluation. Specifically, hand tracking adds to the immersion of the system, placing the user in the first person with full awareness of the position of their hands. Thus, this work addresses the influence of hand tracking in memory assessment with IVR systems. For this, an application based on activities of daily living was developed, where the user must remember the location of the elements. The data collected by the application are the accuracy of the answers and the response time; the participants are 20 healthy subjects who pass the MoCA test with an age range between 18 to 60 years of age; the application was evaluated with classic controllers and with the hand tracking of the Oculus Quest 2. After the experimentation, the participants carried out presence (PQ), usability (UMUX), and satisfaction (USEQ) tests. The results indicate no difference with statistical significance between both experiments; controller experiments have 7.08% higher accuracy and 0.27 ys. faster response time. Contrary to expectations, presence was 1.3% lower for hand tracking, and usability (0.18%) and satisfaction (1.43%) had similar results. The findings indicate no evidence to determine better conditions in the evaluation of memory in this case of IVR with hand tracking.

## 1. Introduction

Virtual reality (VR) can be presented differently depending on the degree of immersion: non-immersive, semi-immersive, and fully immersive. Non-immersive VR offers virtual environments from a conventional computer where users control their movements through a joystick or other control device [1]. Desktop applications have been used in psychological research for decades, although this term is not applied uniformly across studies [2]. On the other hand, to provide the user with a series of experiences similar to real ones, VR has evolved by implementing immersive virtual reality (IVR) environments, offering the perspective of living in a simulated reality [3]. This type of VR promises a wealth of experiences, combining sensations for training tasks and learning new skills, even helping healthy people redesign themselves for a more meaningful and exciting life [4].

An immersive system can consist of concrete surrounding projection surfaces, or even VR glasses, which virtually place the user within the virtual environment for a high level of immersion [5]. A system that uses VR glasses known as a Head-Mounted Display (HMD) is considered immersive since the user sees only the computer-generated image, and the rest of the physical world is blocked from view [6,7]. A total immersion system can incorporate a wide field of view, high resolution, HMD, and auditory, tactile, or force feedback [6,8].

VR systems contain parameters, such as immersion and presence, which are two terms to consider in the user’s performance when executing virtual tasks [7,9]. Immersion is related to technology, while presence is a cognitive, perceptual, and psychological consequence of immersion. On the other hand, presence can be understood as the psychological perception of “existing in” the virtual world [10]. The use of HMD devices could affect the sense of presence and immersion, directly impacting participant performance and motivation. On the other hand, hand tracking is a component that has not been studied in the field of cognitive evaluation and rehabilitation, promising benefits in users’ learning processes [11].

VR-based tools represent an alternative for the diagnosis and cognitive rehabilitation, which can be comparable to conventional procedures [12]. Different studies have endorsed an improvement in cognitive abilities in people suffering from trauma, Alzheimer’s disease, Parkinson’s disease, after suffering a stroke, among others [13,14], also demonstrating benefits for neurocognitive assessment and significant ecological validity [15,16]. VR in treating cognitive processes is a thematic axis that has been booming in recent years. Technological progress, reduced hardware costs, and software support allow new applications to be developed that improve the quality of life of human beings. Cognitive problems can affect all audiences, including young people [17] and people who have suffered previous illnesses [18,19,20]. However, older adults tend to present the greatest affectations, which is why it is necessary to take preventive measures to anticipate this reality [21,22,23].

This work compares two interaction methods with a VR application for memory assessment; specifically, hand tracking is compared versus standard controls; both methods are used throughout training and evaluation. This research’s objectives include analyzing different variables to determine the interaction method that favors the VR experience in memory assessment. This analysis addresses the variables of interest of accuracy in the responses, response time, presence, usability, and satisfaction. The study expects better results for the interaction with hand tracking since this element complements the first-person experience, involving the user more actively. In addition, the naturalness of the movements when selecting an object can facilitate the interaction and, therefore, the memorization of the objects.

## 2. Related Works

Related works are presented in the following order: VR applications for cognitive activities, IVR applications in cognitive activities, IVR studies involving participants without a diagnosis of neurodegenerative diseases, and applications with hand tracking as an interaction method.

VR applications for cognitive activities—The found bibliography shows many applications of VR in cognitive evaluation and rehabilitation. In [24], a virtual environment is presented that allows the person to train sensory memory and space orientation with excellent results. Something similar is described in [25], where a museum is shown, in [26] an airport is shown, and in [27] a virtualized city is shown. Likewise, spatial orientation can be merged with working memory, as in [28], which allows the user to navigate the environment and remember images. Similarly, in [29] a virtual store is presented, and in [30], a town where patients must remember objects. In this application, the working memory, sensory-visual memory, spatial orientation, and executive function are being trained in an integral way.

There are experimental studies that have used VR to assess various cognitive functions. The authors in [31] used a VR application with six daily activities to evaluate 18 patients with Alzheimer’s disease. The cognitive functions examined were episodic memory, prospective memory, visuospatial orientation, executive functions, attention, and general processing speed, finding significantly worse results in the participants with diagnosis compared to a control group concerning the percentages of achievement and the times required to complete the game. The research was also applied a usability questionnaire, where participants found the game easy to use, enjoyed playing it, and had no problems interacting. Another recent study used a virtual supermarket VR application installed on a tablet to assess the cognitive performance of patients with mild cognitive impairment, in addition to measuring brain activity using a portable electroencephalogram and comparing the results with a traditional test [32]. The authors concluded that the experimental tool is helpful for the detection of mild cognitive impairment and cognitive evaluation in an accessible and pleasant way for older adults.

More and more VR-based studies are introducing simulations of ADLs in cognitive deficits. Using these virtual environments has shown significant improvements in general cognitive functioning concerning traditional methods [33]. In [34], the authors found a case in which training in a virtual kitchen allowed a subject with Alzheimer’s disease to relearn cooking activities, and said learning remained stable over time. For its part, the study by [35] found that the cognitive functions of adults with mild cognitive impairment showed greater neuronal efficiency. All this background invites us to think about the need for a methodological framework for designing VR tasks involving ADL with different cognitive impairment levels.

IVR applications in cognitive activities—For several years there has been a growing motivation to develop IVR applications oriented to human cognition to achieve a higher level of immersion [36]. The degree of immersion is important when developing VR applications since it has even been considered a dimension in clinical neuropsychology when evaluating spatial cognition and executive functions. The degree to which participants feel present in the virtual environment has implications for task performance, positively benefiting users’ cognitive performance when recalling facts or memorizing elements [37,38]. A systematic review of studies addressing the efficacy of VR-based rehabilitation reviews the relevant literature on the role of immersion in memory assessment and rehabilitation [39]. This review concludes that there are some theoretical advantages of IVR over non-immersive technology, but there needs to be more evidence to draw definitive conclusions.

IVR studies involving participants without a diagnosis of neurodegenerative diseases—Virtual environments have different immersion levels, so there are divided criteria for the effects produced in the evaluation and cognitive rehabilitation processes. On the one hand, the results obtained by [40] mention that greater immersion offers better performance, while [41,42] describe that there is no significant relationship. Therefore, there is a need to develop more investigations that allow the analysis of the elements that contribute to the immersion of the system. In this sense, hand tracking contributes to the immersion of the system; in this way, it seeks to generate an authentic experience for the user, who, while performing an evaluation, can interact with the environment and feel comfortable. More visual stimuli are offered, emphasizing the emphasis on establishing objects as similar to a real environment. The elements used in the activity are found in a conventional kitchen, contributing to the familiarity of the user while carrying out the diagnosis process.

Regarding the influence of immersion in VR applications for memory training, the question is whether the accuracy of memory retrieval is better in systems with hand tracking compared to the application with controllers. Some works, such as [43,44], have shown significant improvements when the exercises have been carried out in immersive environments. However, changes within this level of immersion still need to be analyzed. In the first case, significant differences were found in the performance of a memory test by the experimental group using IVR. In contrast, in the second study, the results showed an improvement in episodic memory performance in the group that performed the memory exercise in the desktop application condition. Thus, there is still a long way to go to determine whether the degree of immersion guarantees better performance in the different memory tasks.

On the other hand, few studies analyze active navigation in a virtual environment and its effect on memory performance. This includes the user’s physical and psychological activity, that is, motor control and decision-making. In this way, we have the effect of virtual enactment, defined as the influence provided by active navigation or another component on memory retrieval, compared to passive virtual observation [45]. An association or analogy is produced here between the representation effect [46] and active virtual navigation, since both selectively impact specific object processing, such as recognition, but not relational processing. Some studies have shown that this effect can improve spatial memory performance [47] and episodic memory [48]. However, more recent studies did not find significant differences in spatial learning when comparing both navigation methods [49].

Memory-oriented IVR applications have been tested primarily on healthy people and patients with mild cognitive impairment. Another work used an immersive virtual store using the HMD Nvisor ST50 device to assess episodic memory in healthy patients who had to memorize 12 items [30]. The results indicate high levels of presence and motivation, and memory performance on the IVR task is positively correlated with performance on the traditional memory task for both age groups. In addition, the system in this proposal is sensitive to notable age-related differences because older adults omit more items than younger ones. Other authors presented an immersive system using the HMD nVisor ST50 device, with a virtual environment made from a virtual store to assess everyday memory [50]. The participants were 20 healthy young people, 19 healthy older adults, and 35 older people with subjective cognitive impairment who performed the virtual store task. The results obtained with the healthy group allowed researchers to know if the proposal showed adequate levels of difficulty, as well as the effects of the task on age, while the results of the group with cognitive impairment allowed the researchers to relate the performance in the immersive task with the performance of memory in two traditional activities. The conclusions indicate that the accuracy of the responses in the experimental activity was sensitive to age due to obtaining better performance in young people. Furthermore, this performance was correlated with the assessment obtained in the traditional tool, specifically with episodic memory functions and processing speed. On the other hand, in [25], the authors developed a highly immersive application for a virtual museum with two rooms. This was validated with young participants who had to walk through the virtual environment while memorizing the details of each room. The results showed that the participants more easily remembered the objects that had a shorter spatiotemporal distance from each other.

Applications with hand tracking as an interaction method—Regarding works that use hand tracking in cognitive activities, no studies were found in important sources that allow establishing a point of reference for this research. For example, in [51], the authors investigated the user performance in a memory puzzle task using VR. The experiment was carried out with 30 participants under three conditions: hand tracking, controller without haptics, and controller with haptics. The results of this study indicate better performance for hand tracking in solving the task with hand tracking but not better in the initial selection time. On the other hand, in [52], the authors used VR for endotracheal intubation training with the two interaction modalities (hand tracking and controllers) to analyze usability. The results measured in this study indicate that interactions with hand tracking or controllers were not significantly different. Therefore, in this research, an immersive system was used to assess memory and to analyze the influence of hand tracking. Our proposal is based on the application of [53], where four virtualized cabinets are proposed, with objects to be memorized inside. The user must memorize the elements and identify in which cabinet they are located. The parameters analyzed are accuracy, response time, presence, usability, and satisfaction, and better performance for control using hand tracking was established as a hypothesis compared to the traditional method of manual controllers.

## 3. Materials and Methods

There are many innovative proposals for the evaluation of memory, and as presented in the literature, experimental methods involving technology have been contrasted with traditional methods to guarantee their validity. Specifically, VR has raised expectations, promising better tools to diagnose neurodegenerative diseases on time. In this new context, the resources available for the screening process are multiple, including immersion technology such as hand tracking. Although access to these resources is still limited, further growth in VR technologies is expected.

**Figure 1:**
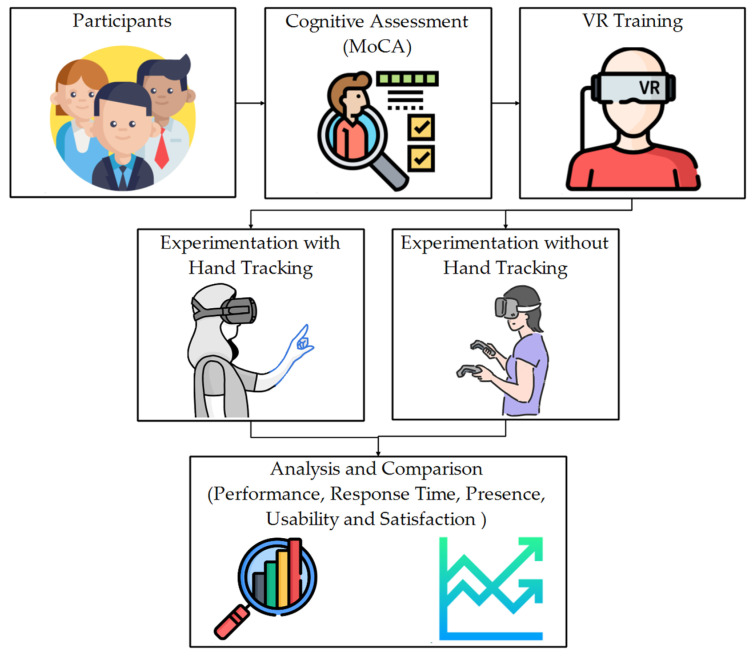
General outline of this research.

The cost of HMD devices has been reduced in recent years, appearing as independent devices that do not require an additional computer for their operation. These options include sensors and cameras that allow scanning of the device’s environment, including hand tracking. This has prompted the development of first-person view VR applications where users interact with their hands [54,55]. Specifically, Meta’s Oculus Quest 2 is a low-cost HMD device that includes this technology as a second option to interact with the virtual world [56]. These features engage users more actively, dispensing additional controls to interact with applications. Although positive effects are expected in the evaluation of memory, no studies contrast this assumption. This work compares the results of a VR application to evaluate using hand tracking with respect to classic controllers. Figure 1 shows the general scheme of the methodology used.

The method begins with selecting participants through a cognitive assessment exam; this allows for the discrimination of participants with low memory performance and for working only with healthy participants. The selected participants are trained in virtual reality to avoid biases related to the lack of experience with the technology used. Next, the memory training and evaluation phases were performed using a VR application with controllers and hand tracking. This application collects data automatically (Performance and response time), and other data were collected manually through instruments (Presence, usability, and satisfaction) for analysis and comparison.

### 3.1. Application

The application designs an activity of daily living (ADL) based on cabinets that have been previously evaluated [53,57]. The activity consists of cabinets and kitchen elements, where the user must remember the location of the elements within the cabinets. In training, the user visualizes the elements inside the cabinets, and the number of elements is configurable according to the required difficulty. The elements are located automatically and randomly inside the shelves, and the doors open for a specific time of waiting for learning. In the evaluation, the user must select the correct cabinet door; this is done with the controllers or the user’s hands, as the case may be. The user has a time limit to choose a door and an element; otherwise, an error is recorded, and the evaluation continues.

Each door conceals a section of cabinet elements and is designed to contain a maximum of 4 elements. In total, four sections are proposed; that is, the capacity is 16 elements. The number of elements can be configured through a menu and corresponds to the required difficulty level. On the other hand, the elements to remember are commonly consumed foods. For this, a database was created with 20 foods: bananas, blueberries, carrots, cereal box, cheese, juice pack, watermelon, mushrooms, skinned chicken, soda can, tomato, yogurt, pizza, kiwi, ice cream, hamburger, ham, cherries, acorn, and cake. A kitchen’s essential components are required for the virtual environment’s design, such as table, chair, stove, glasses, dishes, refrigerator, broom, and clock.

At startup, all the elements of the virtual kitchen are loaded, including the menu that allows user data to be entered and the difficulty level to be set using a laser pointer. The difficulty of the task is limited from 1 to 10, because a larger number of elements is too complicated to remember. When starting the activity, the food is instantiated inside the cabinets, and the training stage begins opening each cabinet door to show the food with a 10 s pause; the doors open one after the other. In the evaluation stage, the question of each element to be remembered is shown. If the wrong door is selected or the waiting time expires (10 s), an error is recorded. On the contrary, if the selection is correct, a success is registered, and each door chosen opens and shows the elements it contains so that the user selects the element in the established time. In the selection of the elements, errors and successes are also required. Depending on the experiment, the user selects the door and the cabinet element using the controllers or the hands.

The training time is 40 s plus the animation time of the doors opening and closing; total, the training time is approximately 65 s. The average evaluation time is 26 s plus the animation time of the doors opening and closing; the average evaluation time is approximately 57 s.

**Figure 2:**
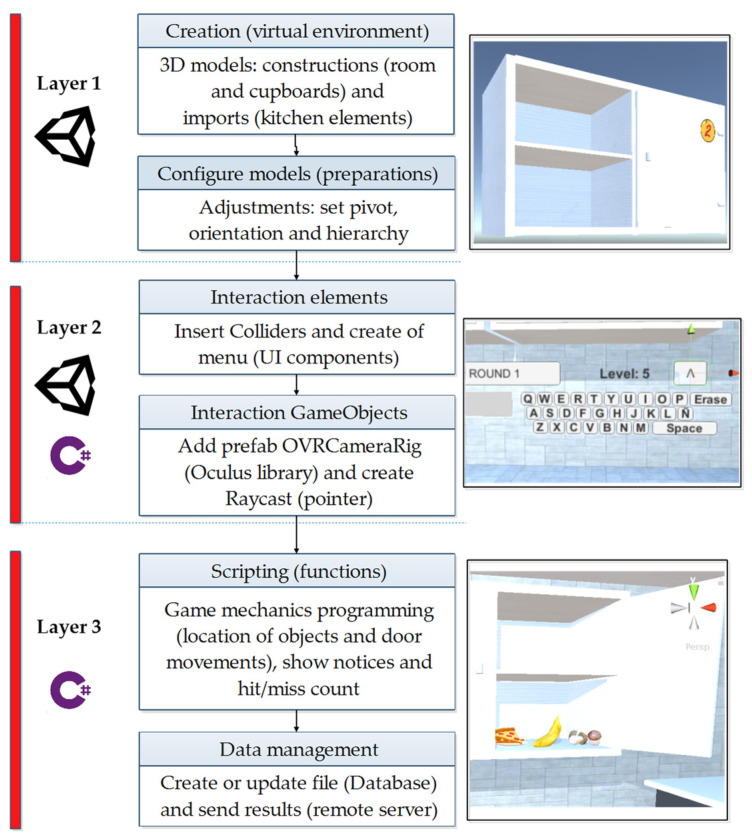
Development layers of the virtual environment.

The virtual environment is built in three development layers as shown in Figure 2—a first construction and configuration layer, then a layer that provides the interaction properties to the environment components, and finally a scripting layer that gives it functionality to GameObjects.

Layer 1—In the creation of the 3d models, the basic construction components provided by Unity are used, using plans, cubes, lights, and others to assemble the room of the scene and the empty cabinets. On the other hand, the more complex elements such as table, chair, dishes, and other kitchen elements are imported from the As-sets of the Unity store, including the foods that are created as prefabs to be instantiated inside the As-sets. cabinets when required. Additionally, it is necessary to prepare these elements to incorporate the movements and other actions of the game; for this, the pivot to the doors is configured, the food is oriented with a view towards the user, and the hierarchies are established.

Layer 2—The interaction of the user with the system components is carried out using a laser pointer, requiring the insertion of a Box Collider component in the cabinet doors and foods, and in this way a collision with the object is detected, which allows the desired actions to be activated. The user menu is laid out within a Canvas with text labels and buttons for entering information, including an alphabetic keyboard for entering the user’s name. To insert the GameObject that represents the tracking camera of the Oculus Quest 2, the OVRCameraRig prefab from the Oculus Integration library was used, which is linked directly to the hardware, and the laser aiming was implemented using a RayCast object that was synchronized with the movements of the controller and hands, as the case may be.

Layer 3—The game procedures are programmed using the rules of the previously described activities, and everything starts when the user presses the start button, destroying and instantiating the food according to the selected difficulty level, opening and closing the doors sequentially with fixed waiting time. Although the activities do not require the locomotion of the player, the automated translation of the main camera is programmed to focus the view towards the middle of each cabinet according to the training sequence. Below are the text labels of the game questions and prompts, and the counters record the successes and failures based on the user’s decisions. Finally, the collected data were grouped into plain text files and sent to the remote server using the SSH:NET library.

### 3.2. Participants

Regarding the participants, healthy candidates with gender equality and with different ages between 18 and 60 years were sought, and all recruited candidates passed the cognitive assessment test. For cognitive assessment, the Montreal Cognitive Assessment (MoCA) test was used, since the group of participants has a high level of education, and only participants who obtain 26 points or more pass. In total, a group of 20 participants was obtained, whose demographics are described in Table 1. Overall, 50% of the participants declare the masculine gender, and another 50% declare the feminine gender. The mean age of the group is 36.05 years with a standard deviation (SD) of 9.69 years, the youngest is 20 years old, and the oldest 56 years old. The mean education time is 19.4 years with an SD of 2.85 years, the shortest time is 15 years, and the maximum is 25 years. In the MoCA test, there is a mean group score of 27.75 with an SD of 1.37, with values between 36 and 30 points.

### 3.3. Procedures

**Figure 3:**
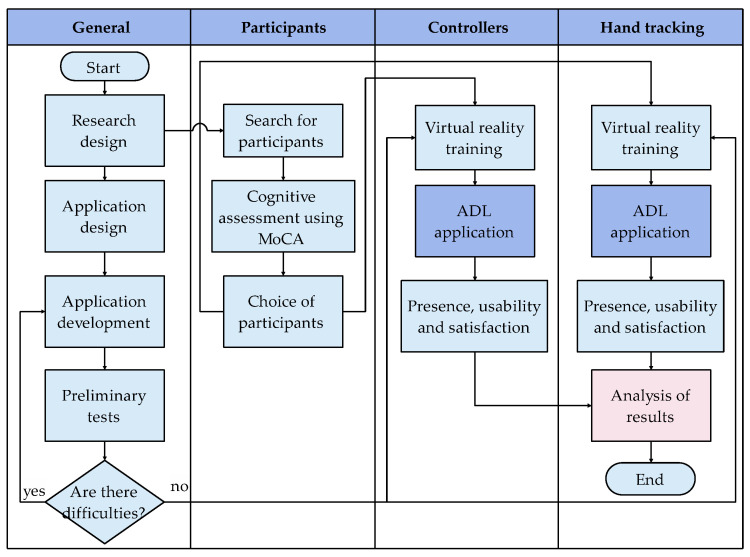
Process diagram of the activities carried out.

All the procedures carried out in this study have the approval of the ethics committee of the Universidad Indoamérica of Ecuador. Figure 3 details the processes to be carried out in this research. To evaluate the proposal, two main activities were designed with a VR application, with the controller and with hand tracking, in that order. Participants must perform a cognitive assessment using the instrument called MoCA, which was done prior to the selection of suitable participants for the tests. On the other hand, the cabinet’s application has a preliminary evaluation phase that allows errors to be corrected before the actual tests. Likewise, the group of participants has a training stage in the use of virtual reality with the Oculus Quest 2 to proceed with the application of the cabinets, at the end of each experimental period the participants complete the presence, usability, and satisfaction test of the application. At the end of the procedures, the results were analyzed and compared.

### 3.4. Instruments

The instruments used to collect data from the participants before and after the experiments with the VR application using the controllers and hand tracking independently are presented below:

MoCA—This simple test assesses multiple cognitive domains, including memory, language, executive functions, visuospatial abilities, calculation, abstraction, attention, concentration, and orientation. MoCA memory tests involve multiple words, some learning tests, a long delay in the recall, and demanding numerical tasks [58]. This instrument was developed in a clinical memory setting and standardized in a highly educated population. Results with 26 points or more are considered to indicate normal cognitive status, and participants with less than 12 years of education have an additional point [59]. In this work, the MoCA test was used as a discriminant to access the experiments as participants because they have a high level of education.

Presence questionnaire (PQ)—This test was used to assess the sense of presence in virtual environments and is made up of 32 questions with seven response levels [60]. The evaluation items consider the factors of control, sensation, distraction, and realism. Only 29 questions were considered in this investigation because the questions related to haptic and sound interaction (Q15–Q17) were discarded [61]. Scores for questions Q14, Q17, and Q18 were calculated by subtracting the value of 7, and scores for all remaining items were calculated by subtracting 1 from the value chosen by the user.

Usability Metric for User eXperience (UMUX)—It is a 4-question instrument used for the subjective evaluation of the usability of an application. The responses have 7 levels of selection, from strongly disagree to strongly agree. The results of this test have been validated with respect to the 10-question usability scale, and they were organized according to ISO 9241-11 [62]. The usability measures provided by this tool allow for a broad understanding of the user experience based on a compact instrument.

User Satisfaction Evaluation Questionnaire (USEQ)—It is a 6-question questionnaire to assess satisfaction with virtual rehabilitation systems. Question Q5 has a negative answer; it must be subtracted from the maximum value plus one to obtain its real score [63]. Although the original tool has a 5-level Likert scale, in this work, a 7-level scale was used to match the rest of the post-experimentation instruments, allowing for the expansion of the instrument’s sensitivity level.

The data are processed through statistics; the significant statistical difference was analyzed using the Student’s *t*-test for paired samples. Since both data come from the same group of individuals, a significance level of 5% (α = 0.05) was established.

## 4. Results

**Figure 4:**
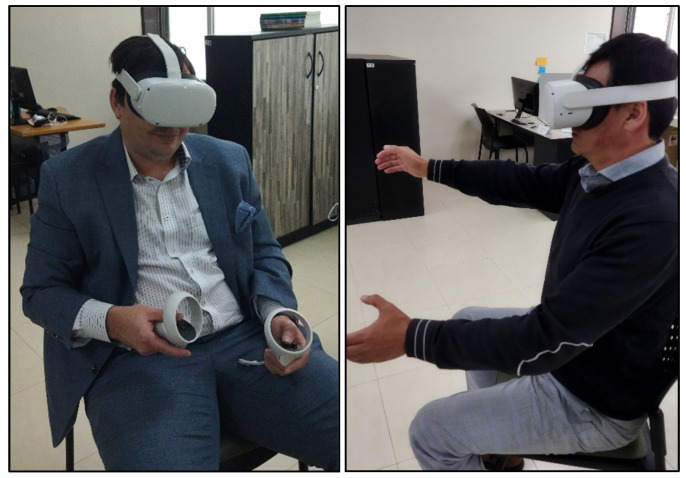
Participants using the application of Oculus Quest 2 with controllers and hand tracking.

The experiments using the VR application based on an ADL were carried out in the following order. On the first session, the application was evaluated using the traditional Oculus Quest 2 controllers, and on the second session, hand tracking was used to assess the application. Our research is based on [64], where the participants used mouse, hand tracking, and controller in VR without altering the order of use of the technology. Although there is research using randomization [55,65], this has not been demonstrated to produce different results. Figure 4 shows photos of the participants using the Oculus Quest 2 with the controllers and hand tracking, respectively. The view of the virtual environment from the user’s perspective was monitored from a phone screen to verify the correct development of the tests. The duration times of each session were approximately 25 min, including data entry, breaks, training, evaluation, and answering tests.

**Figure 5:**
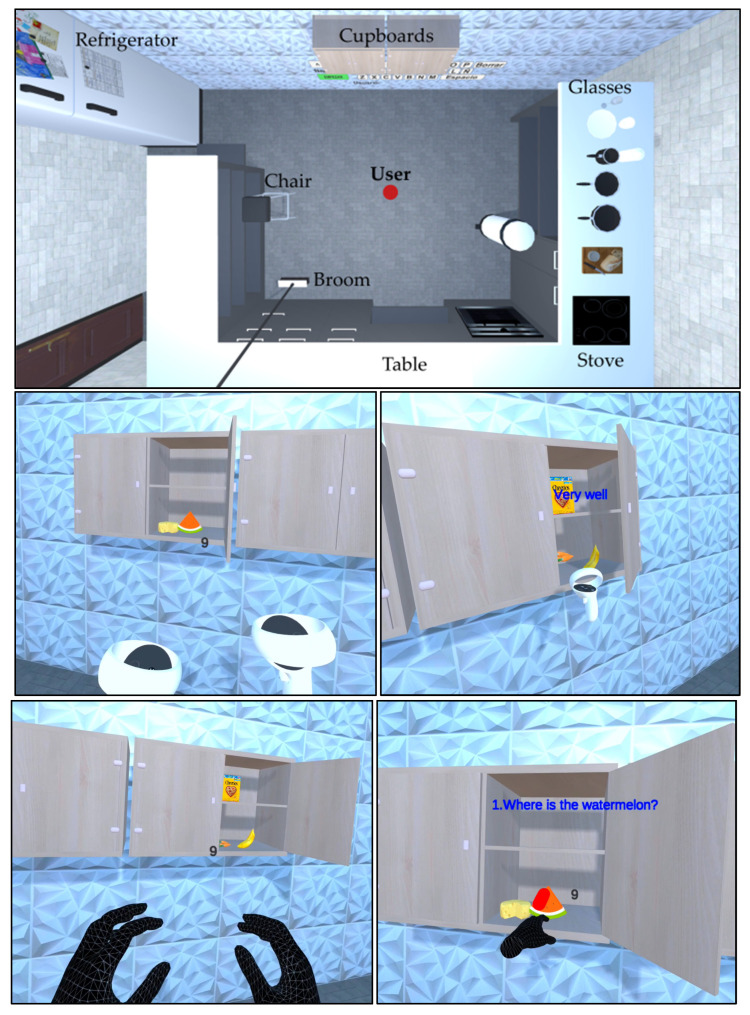
Virtual environment: full scene top view and user view images.

In this research, the application was configured for a difficulty level of 7 elements because classical studies establish a range of 5 to 9 elements as the appropriate number to assess memory [66]. Figure 5 shows images of the virtual environment, observing the whole room setup and the cupboards from a first person view, where the user is in a central position of the room visualizing each element that provides realism to the scene. Users can move freely in the virtual environment; they use locomotion in both stages, in training to better visualize the objects and in the evaluation to get closer to the selected element. When using the controllers, the user only sees the control devices that are synchronized with the real movements, and in the case of hand tracking, the hands are presented according to the user’s movements. In addition, the images show the cabinets, food, and information texts incorporated into the application.

### 4.1. Accuracy

**Figure 6:**
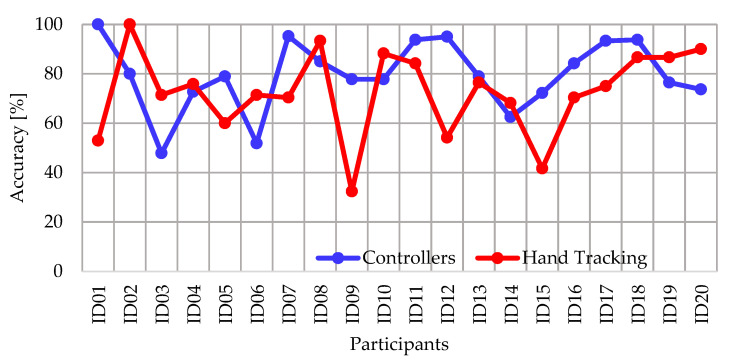
Comparison of VR app activity and the accuracy of the answers.

**Figure 7:**
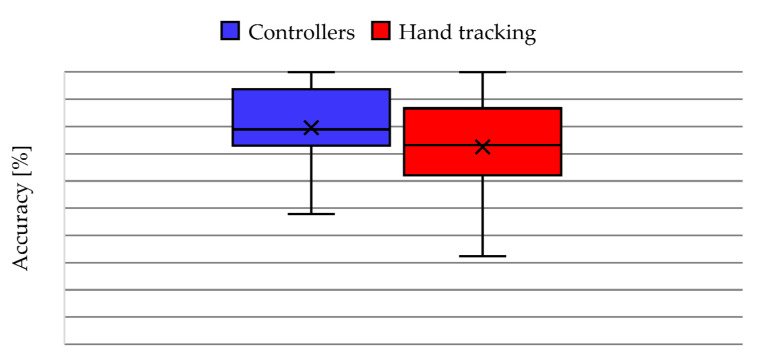
Box-and-whisker plot of accuracy of the answers in VR app activity.

The performance of the participants in the virtual activity was determined from the accuracy of the answers (success/(success + errors)). Figure 6 shows the comparison of the accuracy between both use cases of the VR application (Controllers vs. Hand Tracking), where 11 participants had a better performance using the controllers and 9 participants improved their accuracy when using hand tracking. In addition, the mean accuracy with controllers (U = 79.55%) was higher than the mean with hand tracking (U = 72.47%), which is evidenced in the box and whisker plot of Figure 7. Regarding the dispersion, the accuracy with controllers has a lower SD (13.72%) with respect to the accuracy using hand tracking (SD = 17%). Furthermore, the statistical analysis determines a *p*-value = 0.085.

### 4.2. Response Time

**Figure 8:**
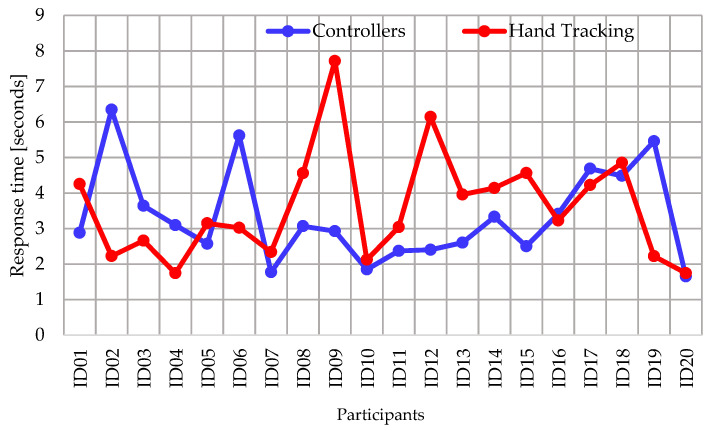
Comparison of response times in VR app activity.

**Figure 9:**
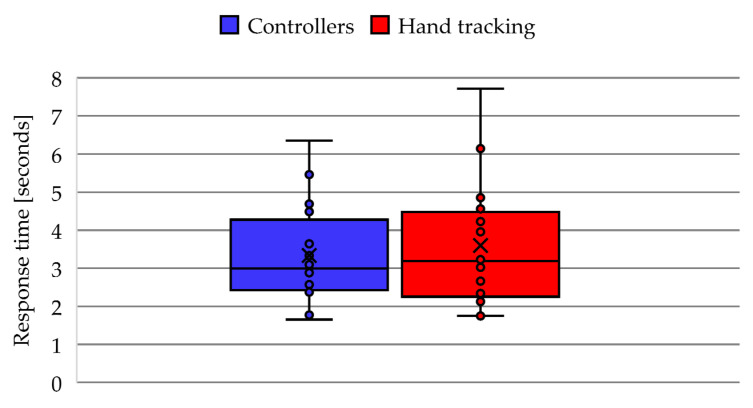
Box and whisker plot of response times in VR app activity.

To analyze the response time of the participants, all the cumulative times of the door selection and the selection of food inside the cabinet were averaged in a complete experiment. Figure 8 shows the comparison of response times between both cases of the activity, where 13 participants had a faster response when using the controllers and 7 participants were faster when using hand tracking. In addition, the mean response time with controllers (U = 3.33 s) was faster than the mean with hand tracking (U = 3.6 s), which is evidenced in the box and whisker plot of Figure 9. Regarding the dispersion, the response time with controllers has a lower SD (1.3 s) compared to the accuracy using hand tracking (SD = 1.5 s). Furthermore, the statistical analysis determines a *p*-value = 0.29.

### 4.3. Presence

**Figure 10:**
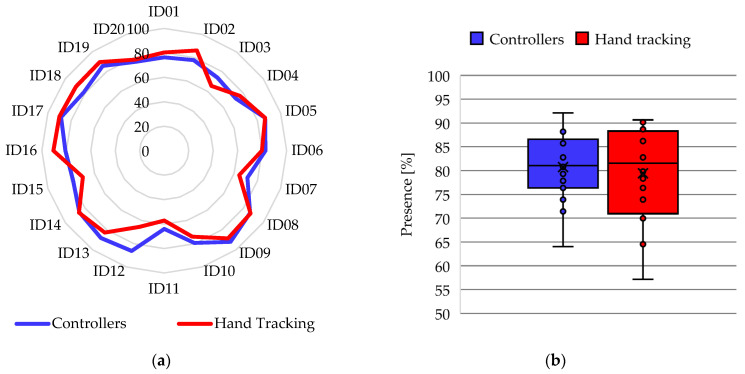
Results of the participants in the PQ instrument: (**a**) Radial diagram of the comparison between the scores of presences in the activity of the VR application; (**b**) Box-and-whisker plot of presence scores in VR app activity.

The results obtained from the PQ instrument were scaled by being converting to percentages to analyze the perception of presence of the participants when using the application with both interaction methods. Figure 10a shows the comparison of the presence score assigned by the participants, where 10 participants indicate a higher score for hand tracking, 9 participants indicate a higher score for controllers and 1 participant has exactly the same score for both methods. In addition, the mean presence score with controllers (U = 80.74%) is slightly higher than the mean with hand tracking (U = 79.43%), which can be seen in the box-and-whisker plot of Figure 10b. Regarding dispersion, the presence score with controllers has a lower SD (6.85%) compared to presence using hand tracking (SD = 9.95%). Furthermore, the statistical analysis determines a *p*-value = 0.21.

**Figure 11:**
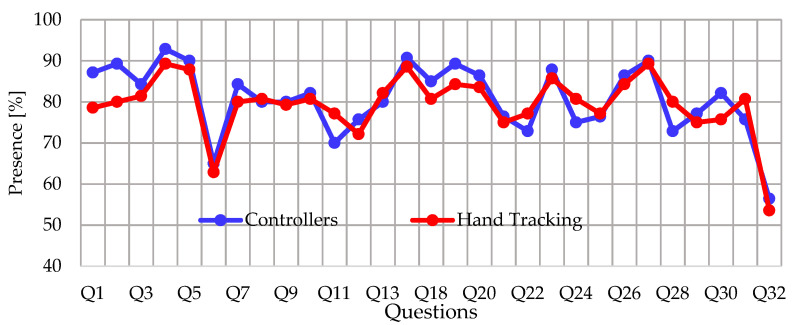
Comparison of the mean scores assigned by the participants in the questions of the PQ instrument when using the application with different interaction methods.

In relation to the items evaluated in the PQ instrument, Figure 11 shows the comparison of the presence scores organized by questions obtained with the mean of the answers of all the participants.

### 4.4. Usability

**Figure 12:**
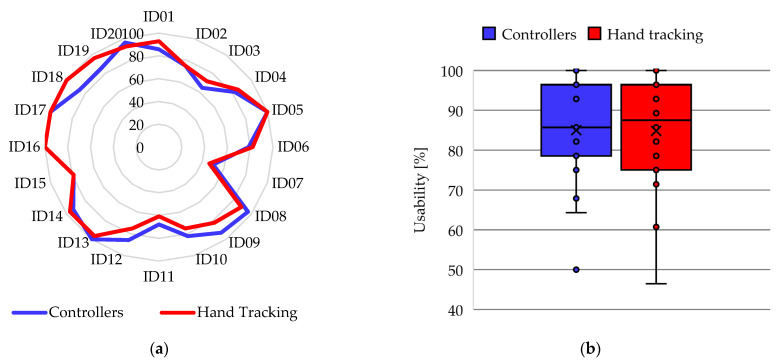
Results of the participants in the UMUX instrument: (**a**) Radial diagram of the comparison between the usability scores in the activity of the VR application; (**b**) Box-and-whisker plot of usability scores in the VR app activity.

The results obtained from the UMUX instrument were scaled by being convertedto percentages to analyze the perception of usability of the participants when using the application with both interaction methods. Figure 12a shows the comparison of the UMUX score assigned by the participants, where 8 participants indicate a higher score for controllers, 7 participants indicate a higher score for hand tracking, and 5 participants have exactly the same score for both methods. In addition, the mean presence score with controllers (U = 85%) was almost the same as the mean with hand tracking (U = 84.82%), which can be seen in the box-and-whisker plot of Figure 12b. Regarding the dispersion, the usability score with controllers has a lower SD (13.1%) compared to the presence using hand tracking (SD = 14.24%), although the former has an atypical value. Furthermore, the statistical analysis determines a *p*-value = 0.45.

**Figure 13:**
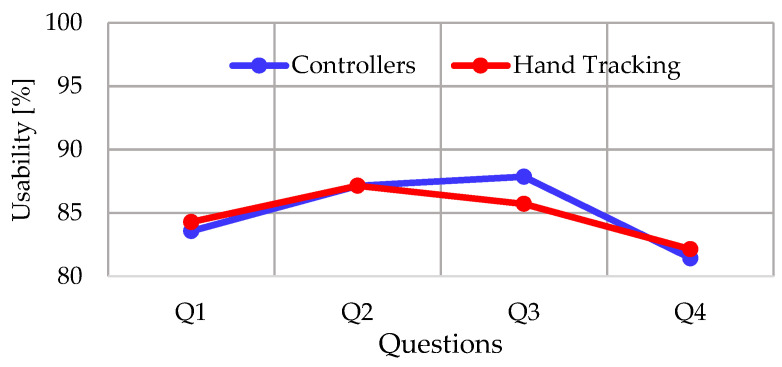
Comparison of the mean scores assigned by the participants in the questions of the UMUX instrument when using the application with different interaction methods.

In relation to the items evaluated in the UMUX instrument, Figure 13 compares the usability scores organized by questions obtained with the mean of the answers of all the participants.

### 4.5. Satisfaction

**Figure 14:**
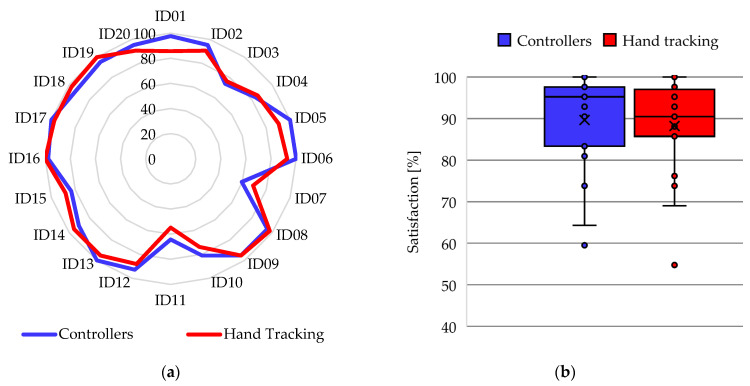
Results of the participants in the USEQ instrument: (**a**) Radial diagram of the comparison between the satisfaction scores in the activity of the VR application; (**b**) Box-and-whisker plot of VR app activity satisfaction scores.

The results from the USEQ instrument were scaled by being converted to percentages to analyze the participants’ satisfaction when using the application with both interaction methods. Figure 14a shows the comparison of the satisfaction score assigned by the participants, where 10 participants indicate a higher score for controllers, 9 participants indicate a higher score for hand tracking, and 1 participant has the same score for both methods. In addition, the mean USEQ score with controllers (U = 89.64%) is slightly higher than the mean with hand tracking (U = 88.21%); this can be seen in the box-and-whisker plot of Figure 14b. Regarding the dispersion, the SD of the satisfaction scores with controllers and hand tracking have similar values (11.6% and 11.36%, respectively), although there are outliers for both cases. Furthermore, the statistical analysis determines a *p*-value = 0.15.

**Figure 15:**
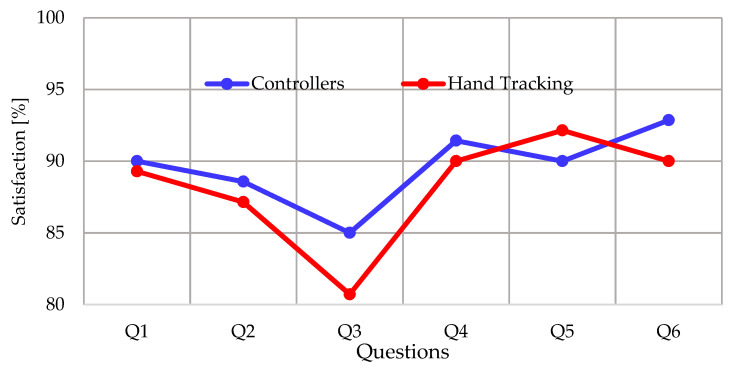
Comparison of the mean scores assigned by the participants in the questions of the USEQ instrument when using the application with different interaction methods.

In relation to the items evaluated in the USEQ instrument, Figure 15 compares the satisfaction scores for each question obtained with the mean of the answers of all the participants.

## 5. Discussion

Our research uses two interaction methods for active navigation without finding a big difference in response precision and response time. Although there is a certain advantage in the mean accuracy of the answers using controllers, similar to [55,64,65], in [67] the users described greater physical and cognitive effort when performing tasks with their hands in the virtual environment. In [51] the results indicate the opposite, obtaining a better performance when using hand tracking in a cognitive task, requiring more studies analyzing user performance with different interaction methods. In our case, a difference of 7.08% was not decisive to establish which was better, and the statistical value indicates no statistically significant difference. On the other hand, in response times, the participants were faster using the controllers, similar to [51]. However, a difference of 0.27 s is not decisive to establish which is better, considering that 35% of the participants were faster when using hand tracking. Furthermore, the statistical value confirms that there is no statistically significant difference. It is evident that studies are lacking clarification on these uncertainties, so future work should incorporate tests with multiple levels of active navigation and evaluate the types of memory to show more explicit evidence about such effects of virtual recreation.

A term closely related to and inherent to the immersion of VR systems is presence. In [68], the authors developed a VR application with two variants for different levels of presence. The group that used the application with the highest level of presence remembered 28% more objects than the other variant of the application, which could imply a positive relationship between the degree of presence and cognitive performance on the test. Other findings raise the question of whether this benefit only occurs in cases where immersion manipulation affects elements of the environment that are directly relevant to the subsequent assessment of memory [69]. Therefore, it is interesting to investigate this question, especially when no clear methodology allows for evaluating the levels of presence in the different VR systems. So far, questionnaires have been designed to obtain these levels [70,71,72]. In our work, the difference in the mean value between interaction methods is minimal (1.3%), which does not allow for determining the best presence experience. Moreover, the statistical value indicates that there is no statistically significant difference. The questions with the greatest difference between interaction methods in the presence rating are Q1, Q2, Q11, and Q30. In Q1, the use of the application with controllers is higher by 8.57%. This question is related to the level of control that the user had with the system, indicating that the participants had less control with hand tracking. In Q2 the controllers are higher by 9.29%, and this question is related to the level of sensitivity of the virtual environment, indicating that the participants perceive the interaction method with hand tracking as less sensitive. In Q11, hand tracking is higher by 7.14%, and this question is related to the consistency coming from the senses, indicating that the participants prefer the sensory perception produced by this method of interaction. In Q11 the controllers are 6.43% higher, and this question is related to how well the user can concentrate on the activities instead of the control mechanisms, indicating that the participants concentrate better using the controllers than with hand tracking. The latter is important because the activity requires concentration to improve the accuracy in the answers, although the difference is minimal, and a certain preference for traditional controls is evident. In general, we found no evidence of a better sense of presence by varying the method of interaction, which should be considered in future work to clarify related hypotheses.

Regarding usability and satisfaction, these works have contributed to the universe of research that has proven the benefits of VR in cognitive assessment [29]. The results were favorable both in the use of controllers and the use of hand tracing for the interaction with the application of this research, since high scores were obtained for the UMUX and the USEQ. In usability, the difference in the mean value between interaction methods was negligible (0.18%), which does not allow us to determine the best usability experience, similar to [52]. Moreover, the statistical value indicates that there is no statistically significant difference. In the analysis of the questions, Q3 has the most considerable difference between interaction methods in usability rating. This question is related to the ease of use of the application; in this case, the application with controllers was 2.14% easier to use than the application with hand tracking. In the literature there is a related work that determined a similar result [73]. In this case, the improvement was 7%.

In satisfaction, the difference in the mean value of the USEQ between interaction methods is minimal (1.43%), which does not allow for determining the best alternative regarding satisfaction. Moreover, the statistical value confirms that there is no statistically significant difference. In the analysis of the questions, Q3 and Q6 have the largest difference between interaction methods in the satisfaction rating. Question Q3 of the USEQ is related to application control, confirming that the user has better control when using the controllers. Something similar occurs in question Q6 of the USEQ; this item is related to the use of this application in memory evaluation; in this case, the application with controllers is 2.86% more preferred for this purpose than the application with hand tracking. Although it is a minimal difference, this implies that the participants prefer to use the controllers to assess memory when performing this cognitive activity. In most cases, the indicators are not conclusive, but there is a slight trend toward using controllers to interact with the application from this research. These findings are supported by [73], where there was greater satisfaction with VR controllers, but its conclusions also indicate that the differences are not significant.

The limitations of this study are the interference effect caused by a possible repetition of objects, the lack of randomization in the experiments, and the absence of a control group. Future work could consider these limitations in their studies. The participants limit this work; future research should address the validation of this tool with larger groups of participants who are older or diagnosed with a neurodegenerative disease. In this way, we will have more evidence to elucidate if the tasks developed can be sensitive in the accuracy of the answers or in the execution times, with the purpose of early diagnosis of neurodegenerative diseases [74] reinforcing the validity of the results obtained. These tools have demonstrated their importance in detecting cognitive dysfunctions that traditional tests have been unable to detect [75].

## 6. Conclusions

This research analyzes the influence of hand tracking in an IVR application developed to assess the memory of 20 healthy participants with homogeneous characteristics. The hardware used was the Oculus Quest 2 standalone HMD device, which has two controllers and the hand tracking function. The application is based on an ADL that has been previously evaluated with respect to traditional methods. The factors analyzed are the accuracy of the answers, the response time, the presence, the usability, and the satisfaction of the participants. Contrary to expectations, in all these factors, there is a certain tendency for worse results using hand tracking as a method of interaction with the application. Still, these results are inconclusive because the differences are minor, and none were found to have a statistically significant difference that supports this assumption. On the other hand, the indicators had high scores that guaranteed the acceptance of this type of proposal as a memory evaluation tool, especially in the satisfaction (USEQ) of the participants, which denotes values close to 90%.

In practice, these results imply that using hand tracking technology in immersive VR applications for memory evaluation does not favor or impair performance, reaction speed, perception of presence, usability, and user satisfaction, although these results should be interpreted with caution. On the other hand, in the literature, only some investigations support and refute the findings of this work, which prevents establishing definitive conclusions with the results obtained in this study. Finally, this work is limited by the number or type of participants, which is why new studies are required with a larger population or participants with a diagnosis of a neurodegenerative disease that complements the results of this research.

## Figures and Tables

**Table 1:** Participant demographics.

Demographics	Value	Demographics	Value
		Age	
Gender:		Mean	36.05
Male	10	SD	9.69
Female	10	Min	20
		Max	56
MoCA		Education years	
Mean	27.75	Mean	19.4
SD	1.37	SD	2.85
Min	26	Min	15
Max	30	Max	25

## Data Availability

The data presented in this study are available on request from the corresponding author.
